# From Brazil to Japan: war, education and circulation of knowledge in the travels of Robert King Hall, 1940-1946

**DOI:** 10.1590/S0104-59702026000100013en

**Published:** 2026-06-29

**Authors:** Adriana Mendonça Cunha

**Affiliations:** i Post-doctoral researcher, Graduate Program in the History of Science and Health/Casa de Oswaldo Cruz/Fiocruz. Rio de Janeiro – RJ – Brazil. adriana@getempo.org

**Keywords:** Education and war, Nationalization of immigrants, Good Neighbor Policy, Second World War, Robert King Hall (1912-1981

## Abstract

This article analyzes the relationship between observations by the American educator Robert King Hall on the national integration of immigrants in Brazil and his proposals for education in postwar Japan. Hall’s 1940 trip to Brazil under the aegis of the Good Neighbor Policy exposed him to Japanese teaching methods, knowledge which equipped him to subsequently participate in educational reforms in occupied Japan. In line with studies on the circulation of knowledge, this analysis addresses Hall’s work on Brazilian and Japanese education, and argues that his experience in Brazil was important for his role as an intellectual in the service of the US government.

In 1940, Robert King Hall (1912-1981)^1^ arrived in Brazil to study the secondary education reforms implemented by the government of Getúlio Vargas (1930-1945). As part of his doctoral work in comparative education at the University of Michigan (UM), he investigated federal control of secondary education in three Latin American republics: Argentina, Brazil and Chile. His visit to Brazil came about through a six-month scholarship granted by the Brazil-United States Institute (IBEU) via the Brazilian Fellowship Program (BFP), an agreement signed between the institute and UM (Kropf, maio-ago. 2020). The agreement was in line with the Good Neighbor Policy, which promoted good relations between the United States and Latin America via economic and political accords and scientific and cultural exchanges (Ninkovich, 1981; Pike, 1995; Sadlier, 2012; Smith, 2017; Valim, Mauad, 2023).

In Brazil, Hall traversed the country visiting schools, giving lectures and establishing contacts with educators such as Lourenço Filho, Paschoal Lemme and Anísio Teixeira. He also collaborated with the National Institute of Pedagogical Studies (Instituto Nacional de Estudos Pedagógicos, INEP) for access to documents and information on Brazilian education. In addition to secondary education, Hall’s attention was captured by the issue of immigrants, who until the early 1940 lived in an isolated manner that preserved the language, education and culture of their countries of origin. Interested in this subject, he visited the states of Santa Catarina and São Paulo to observe efforts by the Estado Novo administration (1937-1945) to nationally integrate foreigners. In São Paulo he witnessed the closure of clandestine schools and came into contact with Japanese textbooks and teaching methods. Concerned that subjects of the Axis countries (Germany, Italy and Japan) were present in Brazil, and the danger these groups could pose for the future of inter-American relations, Hall sent a report to the US embassy with his observations on the German and Japanese colonies.

Upon returning to the United States, Hall mobilized the knowledge he acquired in Brazil to serve the geopolitical interests of his country. Initially, he worked in 1943 with the National Japanese American Student Relocation Council (NJASRC), which helped Japanese American students who had been interned in concentration camps resume their studies (Niiya, 1993). He then coordinated a team of military translators responsible for translating Japanese textbooks. Finally, in 1945, when Japan was defeated and occupied by the Allied Forces, Hall served the US government by working on educational reforms that were implemented there.

This article (the product of my doctoral dissertation, defended in 2024 in the Postgraduate Program in History of Science and Health at Casa de Oswaldo Cruz/Fiocruz) examines the relationship between Robert King Hall’s observations on the national integration of immigrants by the Estado Novo administration and his work in Japan after Second World War. My sources included Hall’s work on Brazilian and Japanese education (his dissertation, articles, books and reports), as well as decrees and newspaper articles reporting on his travels in Brazil. This analysis aligns with studies on the transnational circulation of knowledge, people and practices, contributing to approaches that reveal the non-linear nature of these flows and emphasizing the modifications and constructions that result from these meetings and disputes between the various actors that move within them (Secord, dez. 2004; Raj, 2007; Fan, 2012; Raposo et al. 2014).

In my investigation of how Hall mobilized the knowledge he acquired in Brazil to collaborate with US foreign policy, I argue that his experiences served as a “laboratory” to formulate his educational projects. His Brazilian trip made it possible for him to be invited to work in Japan, decisively contributing to his recognition as an expert in education and his consequent work as a technical consultant in countries like Brazil, Saudi Arabia and Iran during the Cold War.

This article is divided into four sections. The first addresses the negotiations surrounding Hall’s trip to Brazil in 1940, his academic interests, connections established in the field of Brazilian education, and the role of the Good Neighbor Policy in promoting exchanges between Brazil and the United States. Next, I present Hall’s notes on the process of nationalizing immigrants, highlighting the importance of this topic within the wartime context as the US sought to fight the Axis’s influence in Latin America by making Brazil its main ally in the region. In the third section I explore Hall’s work in Japan, discussing his suggestions to reform Japanese education and linking them to his investigations in Brazil. Finally, in the last section I trace Hall’s career after returning to the United States, including new trips to Brazil and regions that were of interest to the US government during the Cold War.

## The Good Neighbor Policy, educational exchanges and Hall’s visit to Brazil

At the Inter-American Conference for the Maintenance of Peace held in Buenos Aires in 1936, US president Franklin Roosevelt expressed his government’s interest in establishing cultural cooperation with Latin American countries via the Convention for the Promotion of Inter-American Cultural Relations, pledging to promote an official financing policy for the region. This measure was in line with the Good Neighbor Policy, which was intended to strengthen ties between the United States and Latin America through economic and political agreements and scientific and cultural exchanges (Ninkovich, 1981; Sadlier, 2012; Smith, 2017).

The adoption of this new strategy marked a change in US foreign policy toward Latin America, which had long been driven by the “big stick” ideology. This transformation resulted from a number of factors, starting with the growth of anti-American sentiment among Latin Americans, especially in Central America and the Caribbean, where several military interventions took place (Schoultz, 2000). The international context, marked by the rise of fascism and Nazism, seemed conducive to a new global conflict. In the case of Germany, the growth of trade relations between Hitler’s regime and countries in Latin America (like Brazil) caused concern in the United States, while the presence of German, Italian and Japanese immigrants who maintained strong ties with their countries of origin and admiration for the German war machine among the armed forces in the region also caused apprehension (Moura, 1980).

The United States then decided to invest in public cultural diplomacy, with cooperation from private institutions that had been active in this area since the early twentieth century (Ninkovich, 1981; Faria, 2002).^2^ In 1938, the Division of Cultural Relations was created within the State Department for interchanges with Latin America through sponsored exchanges with US government resources. Two years later, the Office of the Coordinator of Inter-American Affairs (led by the businessman Nelson Rockefeller) began to organize economic agreements and educational and cultural exchanges, with strong investments in the use of radio, cinema, the press and propaganda (Tota, 2000; Graham, 2015; Valim, Mauad, 2023).

Notably, many countries considered cultural exchanges important for their foreign policy and encouraged the establishment of binational institutes and scientific exchanges (Lessa, 1998; Sá et al., 2009; Silva, 2010). In Brazil, the government made investments to create closer ties within Latin America by establishing cultural missions in several countries in the region (Suppo, Lessa, 2012). Along these lines, binational institutes played a prominent role as spaces for cultural dissemination, expanding contacts between nations. This was the case of the Brazil-United States Institute (Instituto Brasil-Estados Unidos, IBEU); established in 1937, this institution offered English courses and also promoted relations between the two countries by organizing lectures and exhibitions, receiving intellectuals and artists, and through educational exchanges.

Through the initiative of IBEU, an agreement was signed in 1938 between the institute and UM to establish the Brazilian Fellowship Program (BFP) to promote educational exchange between the two countries. The BFP was in line with the Good Neighbor Policy, and served the interests of UM in attracting foreign students to the Midwest while also establishing IBEU on the circulation routes established by inter-American cultural diplomacy. Based on the notion of reciprocity, the BFP established that IBEU would provide three scholarships for American students and UM would grant the same number of fellowships to Brazilians (Kropf, 2020).

Robert King Hall, who was pursuing a doctorate in comparative education at UM, found this initiative to be the ideal opportunity to expand his research on Latin America. He had come into contact with Latin American education prior to the BFP: he investigated secondary education in Argentina during his master’s work at the University of Chicago. He made his first incursion in the region in 1935, visiting nine countries (Panama, Colombia, Ecuador, Peru, Bolivia, Chile, Argentina, Paraguay and Brazil) over three months. His impressions of this trip were featured in an article published in the *Bulletin of the Pan-American Union*, in which Hall (jul. 1936, p.549) argued “that dollar for dollar you can go further, see more, and travel better in South America than in Europe).” He wanted to persuade his compatriots to experience the beauties and cultural richness of Latin America, establishing a relationship of “friendship” with their neighbors.

Brazil, which had not been part of his investigations prior to that time, entered Hall’s trajectory after he was invited to join the trio of students who would come to the country via the IBEU scholarship. From June through October 1940, he visited the states of Amazonas, Bahia, Ceará, Espírito Santo, Goiás, Maranhão, Minas Gerais, Pará, Paraná, Pernambuco, Rio de Janeiro, Rio Grande do Sul, Santa Catarina and São Paulo. During Hall’s research, he conducted interviews and was granted access to information and logistical support from government figures and educators, especially INEP director Lourenço Filho, who not only provided access to sources but also helped Hall define his travel itinerary. Lourenço Filho had himself been in the United States in 1935 to study at the Teachers College at Columbia University, where Hall would work from 1947 onward. Other Brazilians such as Noemy Rudolfer, Anísio Teixeira and Isaías Alves also had contact with American education through trips and studies conducted via scholarships from philanthropic institutions or sponsored by the state departments of education where they worked in the 1920s to carry out significant reforms (Rocha, 2020).

At INEP, Hall was assisted by Paschoal Lemme, who directed the institute’s documentation division and also participated in an IBEU/UM exchange in 1939. Lemme introduced Hall to Anísio Teixeira, who was in Bahia at that time and met him for a conversation (Lemme, 2004). From Bahia, Hall went to Pernambuco, where he was welcomed by Gilberto Freyre in Recife (No Recife..., 3 set. 1940). The two had met in the United States in 1939 when they taught a series of courses and seminars for the Institute of Latin American Studies as part of the University of Michigan’s summer session (Hall, abr. 1939). During this trajectory, Hall gave lectures at the Paraná School of Philosophy, the Bahia State Department of Education, IBEU and the University of Brazil (Conferências, 19 jun. 1940).

In an attempt to establish a relationship between education and political regimes, Hall’s dissertation (entitled “Federal control of secondary education in the ABC republics”) investigated whether the educational reforms carried out in Argentina, Brazil and Chile were “an instrument to provide educational opportunities for youth” or were “producing a social order that will constitute a threat to democracy” (Hall, 1941a, p.4). Hall’s concerns were justified by the wartime context and the threat Nazi Germany posed to the United States in South America and fears that Latin American countries would join the Axis. In Brazil, Getúlio Vargas (who ruled through the dictatorship of the Estado Novo) adopted a position of “pragmatic equidistance” in relation to the conflict, negotiating with the United States as well as Germany, which generated significant mistrust on the part of the US government (Moura, 1980).

Considering Germany as a laboratory of a great totalitarian experiment, Hall (1941a, p.2) argued that the rise of authoritarian governments in Europe had made it clear that “the educational system in its broadest sense was destined to play an exceedingly important part in the new political order” (p.1). In these regimes, education was focused on shaping soldiers and citizens who would obey their leader. He was consequently interested in investigating secondary education reforms, since this education modality served to train young people and the workforce. In Hall’s opinion, a country’s education model was directly linked to its political system, and education that was too nationalist and centralizing could indicate a shift toward authoritarianism. In the case of Latin America, this would pose a direct threat to US hegemony in the region.

But after his six months of travel, Hall came to the conclusion that although Brazil was not a democracy, the Vargas government did not pose a threat to security in the Southern hemisphere. Secondary education, which he criticized for being “too encyclopedic,” “elitist” and “inadequate for the size of Brazil and its regional inaqualities,” was not the main problem with Brazilian education (Hall, 1941a). After all, most of the population was illiterate and never even reached secondary education; the real problem, he concluded, lay in the high rates of illiteracy and concentration of immigrants from Axis countries who refused to integrate into Brazilian society.

It was precisely this question of immigrants that captured Hall’s attention and led him to visit the states of Santa Catarina and São Paulo and get a firsthand look at the Estado Novo’s nationalization activities. His observations and impressions were later organized in a report to the American embassy and articles published in the United States, making him an “expert” in Latin America and more specifically in the organization of the German and Japanese colonies in Brazil (Robert..., 1942).

## National integration of immigrants and the fight against Axis influence in Brazil

From the 1930, the Getúlio Vargas government’s nationalization plan revealed the isolation of immigrants. In addition to linguistic and cultural differences, German, Japanese and Italian immigrants encountered a lack of public schools to educate their children when they arrived in Brazil. These newcomers from countries with strong educational traditions worked to create their own structures to provide education in the regions where they settled (Kreutz, 2000). The organization of these colonies became an obstacle to the Estado Novo, which attempted to impose a national character on education by standardizing curricula, systematizing content and suppressing foreign elements.

In addition to these internal issues that caused concern in Brazil, the imminent global conflict and the presence of unassimilated German, Japanese and Italian immigrants also worried the United States, which feared a Nazi advance in the region. To solve this problem, Vargas initiated a nationalization program through a series of decrees enacted between 1938 and 1942, adopting measures that included prohibiting the use of foreign languages in commercial establishments, limiting the entry of immigrants, mandatory use of Portuguese in education and teaching Brazil’s history and geography in all schools (Brasil, 30 dez. 1938). After creating the National Commission for Primary Education (Comissão Nacional de Ensino Primário) in 1939, the federal government began to build primary schools in partnership with the states which were intended to replace the immigrants’ schools.

Apprehensive about potential Nazi influence, the government took steps to control the production, import and use of textbooks in the country. It also created the National Textbook Commission (Comissão Nacional do Livro Didático), which was responsible for tasks that included examining books and authorizing or refusing their use, encouraging national production of textbooks, recommending foreign textbooks for translation and promoting national exhibitions of those texts which were authorized for use (Brasil, 30 dez. 1938). It was feared that foreign books could fuel separatism and contempt for Brazilian institutions.

In this sense, the Ministry of Education and Public Health and the Ministry of War acted together: the former was responsible for organizing public education, while the latter investigated and addressed any reactions to the nationalization process, ensuring national security, closing clandestine schools and fighting Axis sympathizers. This process was marked by arrests, political persecutions and indiscriminate acts committed under the justification of war (Seyferth, 1999). At the same time, a number of groups clandestinely sought to preserve the ethnic schools.

Attentive to the immigrant problem, the US conducted investigations to assess the danger these groups posed to Brazil and the Americas (Tota, 2000). For this reason, it was not unusual for Hall to have sent a report to the US embassy in August 1940 describing what he saw on his visits to German and Japanese colonies during the time he spent in the country. This document is strong evidence that Hall’s research in Latin America had not only an academic bent but also a clearly political dimension which was directly associated with his country’s interests in the region.

The topic of foreigners received little attention in Hall’s dissertation, since his research focused on secondary education reforms. But his report to the embassy revealed a careful look at the problem of nationally integrating the Germans in Santa Catarina and the Japanese in São Paulo. Some of the positions stated in this document were later highlighted in the articles he published after returning to the United States. These observations are important to understand not only how the settlers from the Axis countries lived and organized themselves, but also how the Vargas government combated this influence and to what extent it threatened security in the Americas.

This question appears in Hall’s (1941b) article entitled “Foreign colonies of Brazil: a North American view,” where he offered a “neutral” view of the conflict between Brazilians and foreigners in the process of national integration. His analysis begins with the closure of the schools, which in his view was responsible for “a great deal of unnecessary friction and trouble” (p.13). He noted the Brazilian government’s violent approach to immigrants in São Paulo, Santa Catarina and Paraná. On the other hand, he stated that “the Brazilians are correct when they say that the schools were flooded with Nazi propaganda” (p.13). Hall pointed out that in many clandestine establishments, Nazi propaganda materials were found that indicated “the necessity of *Lebensraum* [vital space] for the German people, the superiority of the German army, and the location of Nazi organizations and German colonies in various parts of the world” (p.14). However, he noted that not all Germans necessarily were followers of Nazism or shared these theories, and that the violent actions on the part of the government were unfair. His criticism was not directed at the policy of nationalization itself, but toward the aggressions committed during the process.

Hall recognized that Brazil lived under a dictatorship characterized by censorship and persecution of the regime’s opponents. This point is more evident in the report, when he directly critiqued the Vargas regime. While his articles exhibited a conciliatory posture, his report openly criticized the work of the Brazilian inspectors, which he categorized as “technically ignorant.” According to Hall (14 ago. 1940, p.3), one reason behind the conflict between Germans and Brazilians was the fact that many involved in the nationalization process were clearly negroides from northern Brazil, exercising activities on white and pure Germans with a lot of racial awareness. In another passage, Hall stressed the difficulty faced by the Brazilian government “in convincing Germans to change a strong and recognizably superior culture that had existed for three generations” (p.4). We can see that he was willing to express racist sentiments when addressing the US government representatives, revealing a prejudiced view of Brazilians who he considered inferior to the Germans with their “superior” culture.

Another point Hall noted (1941a, p.180) was the government’s control of foreign books circulating in the country, demonstrating its sharp response to the issue. He stated that activities like these were carried out without selective criteria, in many cases resulting in the confiscation of foreign works that had no connection whatsoever with ideologies like Communism or Nazism. Censorship also impeded the circulation of American books, which to Hall made no sense considering the friendly relationship between Brazil and the United States.

The damage was not limited to books, but also to the spread of American ideas, language and culture among Brazilians. In another article, “English teaching in Argentina and Brazil,” Hall (1942) addressed English language teaching in these countries. In Brazil, his objective was to demonstrate how the Estado Novo’s repressive policy toward foreigners limited the spread of the English language in the country. Hall believed that Brazilians were very interested in the American culture (p.79), and addressed the obstacle facing books only in political terms, as the Brazilian government’s antagonism with regard to foreign languages increased with the discovery of clandestine establishments.

Hall considered these measures unfair, since relations between Brazil and the United States were based on “sincere intentions.” As good neighbors and allies in the fight against the Axis, Americans should not be subject to the same treatment as the Germans and Japanese. Hall (1942, p.81) also highlighted the growing “popularization of English and American culture” among Brazilians as another justification for proximity between the two nations.

Among the Japanese, Hall (1941b, p.18) perceived a strong reluctance to national integration, which he attributed to significant cultural differences compared to the Brazilians. These opinions are clearer in the report, where he indicated that a “clash of races” existed in the relationship between the Japanese and Brazilians, since both sides had very negative views of the other. While the Brazilians considered the Japanese “dirty” and “untrustworthy,” the Japanese viewed Brazilian education as “mediocre” and the authorities “expected bribery” (Hall, 14 ago. 1940, p.8). In Hall’s assessment, these differences made it very difficult for the Japanese to assimilate, and he argued that it was more pertinent to “end all new immigration” (p.12).

His belief in the Japanese inability to assimilate was shared by many Brazilian intellectuals and politicians, who had already discussed this issue long before the war. The press and scientific discourse helped spread this vision, classifying Europeans as ideal immigrants to the country while Africans and Asians were thought to be completely alien to Brazilian culture (Shizuno, 2010; Morais, 2011).

Japanese immigrants first arrived in Brazil in 1908 to work on coffee plantations, and subsequently spread throughout the interior of São Paulo and Paraná. In these regions, they developed colonies where they sought to preserve *yamatodamashi,* Japanese cultural values and patterns (Cytrynowicz, Cytrynowicz, 2017). The combination of prejudice and linguistic and cultural differences caused them to remain isolated, remaining Japanese while living in Brazil (Tanno, 2008). With the arrival of war, bias led to violence and restrictions. In São Paulo, the state government followed federal decrees promoting evictions, surveillance and control of the Japanese. The closure of schools was not accepted by the colonists, who resisted and maintained clandestine establishments. Beyond just instruction, the Japanese residents saw these schools as places for learning the essential values of *yamatodamashi* (Morais, 2011).

Hall pointed out the maintenance of these values, the construction of closed colonies that were well organized in economic, social and cultural terms, and the educational requirements of the Japanese as elements that hindered national integration of this group. In his report, he noted that even when they enrolled their children in Brazilian schools, the Japanese kept them in clandestine establishments that operated late at night or early in the morning. He reported that he “was present when one of those schools was invaded and 27 students and two teachers were taken at 11:30 p.m” (Hall, 14 ago. 1940, p.8).

After analyzing material seized by the police, Hall (14 ago. 1940, p.7) identified the use of teaching materials containing “maps and propaganda graphics” demonstrating the “military force of various countries, Japanese flags, photos of the emperor and of men who died as a human bomb.” As for the seized textbooks, Hall highlighted the presence of elements such as photos of “airplanes, machine guns and flags of Japan, Germany and Italy.” Attached to the report were copies of books seized during a police operation in the city of Marilia, São Paulo. Several of the elements indicated by Hall can be identified: they are written in Japanese, and contain many photos of military figures and flags. The content was taught using elements of Japanese history and culture, in order to glorify the country.

These observations were later useful for the US government, which began the process of occupying Japan after Second World War. In this sense, the knowledge Hall acquired in Brazil was decisive not only for him to be recognized as an “expert,” but also for his role in reforms of the Japanese educational system. His research, which initially focused on secondary education, took on new dimensions from his visit to Japanese colonies. This was only possible because Hall was able to establish contacts with Brazilian educators and institutions that allowed him to closely follow the process of nationalization. This case demonstrates, in the processes of circulation, the way in which we can identify how a traveler belongs to a given social configuration and note what gives meaning to what he collects, while we also notice how the traveler integrates new interests from encounters that occur along the way (Raposo et al., 2014).

## Knowledge in circulation: Hall’s work in Japan (1945-1946)

After the attack on Pearl Harbor in December 1941, Hall’s knowledge of the Japanese colonies in Brazil became useful to the war effort, considering that very little was known in the United States about Japanese culture and values (Benedict, 2014). In 1943, he was assigned to work at NJASRC, which was created to assist Japanese American students who had been interned in concentration camps return to university (Niiya, 1993). During this period, Hall had enlisted in the Navy and attended the Naval School of Military Government and Administration at Columbia University, a school focused on training the officers who would work in the Pacific (Wallace, 1944). In 1945, he moved to Monterey, California, where he worked as head of the Education Division of the Occupation Planning Staff at the Civil Affairs Staging Area, leading a group of military personnel responsible for translating Japanese textbooks (Hall, 1949a).

After the war, Japan was occupied by the Allied Forces under US General Douglas MacArthur, the Supreme Commander for the Allied Powers (SCAP). Yet again, Robert King Hall’s trajectory followed the pathways of American foreign policy, leading him to work on reforms for the Japanese educational system. In Japan, he was appointed chief of the Education Sub-Section of the Civil Information and Education Section of the Civil Information and Education Division (CI&E), an agency responsible for assisting the Ministry of Education in the reform (Hall, 1949a, p.4). The CI&E’s activities included supervising school curricula and evaluating textbooks (Nishi, 1982).

Prior to the war, Japanese education was guided by Confucian principles and the promotion of nationalism and ultranationalism. For this reason, educational reform was seen as indispensable if the United States wanted to demilitarize Japanese society (Mello, 2006). To eliminate nationalism and militarism, SCAP eliminated *shushin* courses (addressing moral and civic discipline, taught in the primary grades) and invested in liberalizing education, removing military officials from schools and militarist content from textbooks (Mello, 2006).

In Hall’s assessment (1949b, p.5), “a peaceful Japan is the Pacific key to world peace, and the re-education of the people is the key to peaceful Japan.” The question was how to make that country, with its culture, religion and values so different from that of the Americans, adopt democracy as a political system. In his opinion, the answer lay in the educational system that the Japanese would adopt after the war, and for this reason it was essential to understand how education worked in the country to help rebuild the Japanese educational system and reform the Japanese political structure (Hall, 1949b, p.8).

In examining Japanese history, Hall (1949b, p.33) was impressed by Japan’s educational program, marked by mass education and significant investments in human resources. He also recognized the country’s ability to insert different educational models, mix them together and adapt them to national values and interests. At the same time, he indicated the dangers and consequences of centralizing education in the hands of the *Monbusho* (Ministry of Education).

The *Monbusho* was responsible for the “control over the selection, training and supervision of teachers, the content of curricula, the preparation of textbooks and the censorship of trade books” (Hall, 1949b, p.33). This ministry was also responsible for maintaining national unity through “teaching of a body of ethical and moral concepts” and *shushin* courses that promoted “surveillance and control of thoughts.” Hall’s criticism of the Japanese education system and Ministry of Education addressed this latter point, marked by the transformation of ethical principles into mechanisms of social control.

In 1937, the *Monbusho* published a document entitled *Kokutai no Hongi* (Cardinal Principles of the National Entity of Japan) containing guidelines for *shushin* courses and drafting textbooks. Hall first came into contact with this material in August 1940 in Brazil, when he accompanied a police operation in a clandestine school in the city of Marilia. What caught his attention was the presence of the *Kokutai no Hongi* as a type of “manual” for the education of Japanese settlers (Hall, 1949a, p.25-26).

According to Hall (1949b, p.71), this document had a decisive influence on Japanese education, was widely reproduced and circulated, was required reading for high school teachers and students and regulated the teaching of ethics of schools. The importance attributed to the *Kokutai no Hongi* is visible in the fact that SCAP’s first act was to suspend *shushin* courses in Japanese schools.

In *Education for a new Japan*, Hall (1949c) discussed SCAP’s first actions in reforming Japanese education, offering the following assessment: “The Japanese educational system remains as potentially dangerous as it was before Pearl Harbor” (p.202). The main reason was the greatest point of contention between Hall and the occupying forces: the reform of the Japanese writing system. In a memorandum entitled “The exclusive use of katakana as official written language,” sent to the Civil Affairs Division of the Department of War on June 23, 1945, Hall recommended banning the use of *kanji* and making the exclusive use of *katakana* mandatory (Unger, 1996).

The Japanese writing system consists of three types of characters: two phonetic “alphabets” known as *hiragana and katakana,* and Chinese characters called *kanji.* Each type has different characteristics, purposes, and ways they are used. *Hiragana* is used for native Japanese words and grammatical elements, and forms the foundation of the language, while *katakana* is used for words and names of non-Japanese origin, loanwords and onomatopoeic expressions. *Kanji*, in turn, is Chinese in origin and considered the most difficult to master; over ten thousand characters exist, and each *kanji* can have more than one meaning. Japanese can also be expressed in written form using the Latin alphabet characters (*romaji*, in a process known as romanization), which is often used by foreigners who are not familiar with the Japanese writing system (Ota, 2001).

Hall suggested eliminating the Chinese-derived *kanji* and using only *katakana*, which was used for foreign words and names and consequently less commonly used by the Japanese. In his opinion, the excessive difficulty of Japanese writing was an obstacle to the development of democracy, thus requiring linguistic reform. In Hall’s assessment, “the written form of Japanese is simply too inefficient to be continue if Japan is to compete on a basis approximating equality with Occidental nations” (Hall, 1949c, p.300). He argued that the Japanese themselves accused the written form of the language of constituting “a major barrier to the reconstruction of the educational system and to the democratization of the country” (p.312). The difficulties involved in learning Japanese writing, according to Hall, revealed a kind of intellectual elitism, since generally only the wealthiest people were able to pursue their studies and take the time required to master the written language (p.312).

Upon arriving in Japan, Hall discovered that Japanese linguists had already proposed reforms of the writing system. Since the establishment of the first National Language Research Council in 1909, scholars had tried to limit the number of Chinese characters used, but the ultranationalism that prevailed prior to the war hindered such movements, which only regained force with the occupation. According to Gottlieb (1998, p.71), many experts used “the new concept of democracy imposed by the Occupation forces to provide a powerful argument in favor of rationalization of the way Japanese was written.” Japanese advocates of these changes allied themselves with some Americans who, like Hall, saw the complex writing system as an obstacle to democratization of the country (Krumrey, 1993). This group included radical reformers who defended total elimination of *kanji,* as well as those with more moderate positions who proposed limiting the number of characters (Gottlieb, 1998).

In contacting Japanese experts, Hall used the local debate to consider ways to facilitate what he viewed as Japan’s transformation into a democracy in line with American ideals. At the same time, Japanese scholars used the rhetoric of democracy to propose reforms they had long defended (Gottlieb, 1998). According to Tsuchimochi (1993), after Hall arrived in Japan and encountered local discussions, he changed his plan to suggest the use of *romaji* instead of his original proposal to exclusively use *katakana.* Along with Jiro Aramitsu, head of the Department of Textbooks, Hall proposed romanizing Japanese textbooks because this would make it “easier for foreigners to read in Japanese” and “easier for the people to read the laws and newspapers and become more literate” (Tsuchimochi, 1993, p.27).

This situation indicates the number of agents and interests involved in transnational processes of knowledge circulation, and shows how local agents in many cases were not only aware but also benefited from the foreign presence to advance their own agendas. As Kapil Raj (2007) notes, circulation is a movement of encounters marked by interactions between actors and heterogeneous groups which promotes the formation of networks and the production of new knowledge within the circulation routes themselves.

In his proposed reform Hall defended American interests, aiming to transform Japan from an enemy into its main ally in Asia. In his view, the Japanese writing system was a “barrier to international understanding and amity,” as “the excessive difficulty of the writing system made Japanese culture relatively inaccessible to Occidentals” (Hall, 1949c, p.312-313). This hampered SCAP’s activities, since “the masses of people were not adequately reached by the information services of the occupation authories” (p.313). Besides the fact that official documents were originally written in English, Hall pointed out that translating them into Japanese “was considered difficult, if not impossible” (p.313). In other words, he proposed a reform that would facilitate the process of occupation in Japan through structural changes in not only the country’s political system, but also its language and culture.

Hall’s proposals generated numerous debates involving members of the US educational mission, Japanese experts, and officials from SCAP and the State Department. However, like the proposal to ban the use of *kanji,* the suggestion to romanize textbooks was not approved. This was decided by CI&E Education Division head Harold G. Henderson, after a discussion with Minister of Education Tamon Moeda, and led to friction between Hall and other members of the division. General MacArthur later made some changes in the agency, and Hall left the textbook division for the planning division, and ultimately transitioned away from functions related to the Ministry of Education (Tsuchimochi, 1993, p.28).

## The Cold War and new circulation routes

In late 1946, Robert King Hall returned to the United States, and became chair of the Comparative Education department at Teachers College at Columbia University the following year. While the knowledge he acquired during his trip to Brazil was crucial to his participation in the educational mission in Japan, his experience in Asia ultimately consolidated his name as a reference in Latin American and Japanese studies. The research Hall conducted and the contacts he established in the field of Brazilian education were fundamental to make him a specialist in comparative education with a trajectory inextricably linked to American geopolitical interests.

In 1948, he returned to Brazil to “visit Brazilian educational leaders he had not seen since the war” and resume his research on education there (Interesse..., 13 jun. 1949). His attention turned to industrial and rural education, priority areas for the Point IV program, a technical assistance plan created by the Truman government which focused on Latin America. Through the signing of bilateral agreements, the Point IV program promoted projects in the areas of economics, administration, agriculture, transport, health and education (Damasceno, 2022). Hall would become a great advocate of the program and US interest in “helping” its Latin American neighbors.

Hall was hired to work at INEP from 1949 to 1951, providing technical advice to the rural education program it coordinated. His activities included training primary school teachers and producing an analysis of the project (Cunha, 2018). Hall’s hiring demonstrates his recognition as an “expert,” and also points to the lasting links he constructed in the field of Brazilian education during his 1940 trip, which lasted into the Cold War context.

However, his presence was not enthusiastically received by everyone. Paschoal Lemme, who helped Hall in 1940, openly criticized his hiring by INEP. After attending a lecture by Hall in Rio de Janeiro in 1949 on the Point IV plan, Lemme noted the “tone of near-arrogance with which the American educator expressed himself, despite his politeness in personal dealings.” To Lemme (2004, p.25), by defending the “good intentions” of the US government in providing technical assistance to underdeveloped countries, Hall was serving as a “spokesman for American imperialism.”

The views of both educators expressed the context of the Cold War, marked by tensions between anti-Communists and anti-Americans around the world. According to Lemme, Hall argued that through the Point IV plan, the US was prepared to “consider technical and financial assistance to the governments of economically underdeveloped countries in order to help boost their standards of living” (Hall, 1949, p.48 cited in Lemme, 2004, p.25). In this “aid,” Lemme saw a mechanism for exploiting poorer countries and maintaining them as “simple producers of raw materials” (p.25).

We can note that throughout his trajectory, Hall’s position in defense of “democracy” takes on new meanings. While within the context of war he criticized Nazism and the strong centralization of the Brazilian educational system, after the conflict he took a position to combat communism and defend the American model. This is evident in his text “Educación para el desarollo económico (“Education for Economic Development”), the product of a lecture given in Buenos Aires in 1950. On the topic of the Point IV plan, Hall (1950, p.560) argued that “the spirit of helping the less fortunate neighbor is always very strong, particularly in the people of the United States.” For this reason, accusations that his country had imperialist interests were “unfounded,” and he reinforced the “goodwill” of “industry and government” in collaborating on the development of other nations (p.561). Despite presenting himself as an “observer completely devoid of official character,” which guaranteed him an “impartial view,” at that time his connection with American geopolitical interests was already quite clear.

From 1945 onward, Hall began to work as an advisor to governments and private institutions, serving as a consultant on educational projects. For example, in 1949 he was hired by the Iranian government to assist in educational reforms, where he recommended adopting the industrial teaching model used in the schools in Brazil’s National Industrial Learning Service (SENAI). And in 1955, he left Columbia University to coordinate staff training at the Saudi Arabian Oil Company; he remained in Saudi Arabia until the 1960s.

## Final considerations

We can state that Hall’s recognition as a specialist, which came about thanks to transnational networks of cultural diplomacy, led him to serve in important positions as a collaborator or work on US government projects, which in turn converged with those of other countries he visited. During these trips, which were defined (although sometimes in unforeseen ways) by the Second World War scenario, Hall participated in transnational academic and political circuits as he passed through geographic spaces which were strategic for the construction of US hegemony.

His case helps explain the importance of the exchanges brought about via the Good Neighbor Policy to the circulation of ideas and educational models in the Americas. As historiography has also pointed out, Hall’s trajectory allows us to observe the complexity, multiple agents and interests, and numerous contingencies surrounding the processes of knowledge circulation between countries.

Hall’s studies on Latin American education were essential for his positioning in the field of US education at a time when pan-Americanism was central to US foreign policy. He was only able to become part of the “Good Neighbor” circuits thanks to increasing government investments in exchange and cooperation policies intended to bring the United States and Latin America closer, fight Axis influence and promote inter-American “friendship.” In this way, Hall benefited from the opportunities that resulted from the Good Neighbor Policy while also directly contributing to the interests that guided it.

His observations of immigrants in Brazil (especially the Japanese) and the Vargas government’s measures to nationalize them led Hall to occupy important posts in the immediate postwar period. This situation was particularly interesting as an illustration of how circulation routes are often constructed through unforeseen contingencies. At the same time, Hall’s trajectory helps us understand the complex and multiple nature of interests and agents involved in the construction of American public cultural diplomacy within the context of the Second World War, when Latin America served as a “laboratory” for the development of US foreign policy, as well as the transformations in this region during the Cold War, taking on new directions and interests that also impacted his objects of study and work.

## Data Availability

Not deposited in a data repository.

## NOTES

1 Robert King Hall was born in Kewanee, Illinois, the descendant of Irish Presbyterians who emigrated to the United States in the late seventeenth century. He studied at the Deerfield-Shields High School and graduated with honors in physics and mathematics from Lake Forest University in 1934. He obtained a master’s degree in physics from Harvard University (1935) and another in education from the University of Chicago (1936), as well as a doctorate in comparative education from the University of Michigan (1941). From 1936 to 1941 he taught at the Cranbrook School, a private boys’ school in Michigan. When the United States entered Second World War he enlisted in the Navy, attended the Naval School of Military Government and Administration at Columbia University, and then served as assistant academic director of the Naval School of Military Government at Princeton University. During 1945-1946 he participated in a mission in Japan in the service of the US government, and joined the faculty of the Teachers College at Columbia University in 1947. As a technical consultant, he collaborated with Brazil’s National Institute of Pedagogical Studies (Instituto Nacional de Estudos Pedagógicos) from 1949 to 1951. In 1949 he served as an advisor to the government of Iran, and in 1955 began to train employees of the Arabian American Oil Company. He returned to the United States in the 1960s and continued to serve as a technical consultant for companies and institutions including the Saudi Arabian College of Petroleum & Minerals.

2 In the early twentieth century, especially after First World War (1914-1917), several philanthropic institutions such as the Carnegie and Rockefeller foundations sponsored scientific and cultural exchanges as a way to promote international relations. It was believed that mutual understanding could make it possible to maintain peace among nations. For example, see: Batista (2024); Batista, Ferreira (2021); Palmer (2015).
