# Supplementary material for: From Brazil to Japan: war, education and circulation of knowledge in the travels of Robert King Hall, 1940-1946
Source: Hist Cienc Saude Manguinhos. 2026 Jun 29;33:e2026013. doi: 10.1590/S0104-59702026000100013en (PMC13317118; doi:10.1590/S0104-59702026000100013en)
Supplement: Translation

# Translation: Do Brasil ao Japão: guerra, educação e circulação de conhecimento nas viagens de Robert King Hall, 1940-1946

**DOI:** 10.1590/S0104-59702026000100013

**Authors:** Adriana Mendonça Cunha

**Affiliations:** i Pesquisadora em estágio pós-doutoral, Programa de Pós-graduação em História das Ciências e da Saúde/Casa de Oswaldo Cruz/Fiocruz. Rio de Janeiro – RJ – Brasil. adriana@getempo.org

**Keywords:** Educação e guerra, Nacionalização dos imigrantes, Política da Boa Vizinhança, Segunda Guerra Mundial, Robert King Hall (1912-1981

## Abstract

O artigo analisa a relação entre as observações do educador estadunidense Robert King Hall sobre a nacionalização dos imigrantes no Brasil e suas propostas para a educação japonesa no pós-guerra. Em 1940, no contexto da Política da Boa Vizinhança, Hall viajou ao Brasil, entrando em contato com os métodos de ensino japoneses. Os conhecimentos adquiridos na viagem o levaram a atuar nas reformas educacionais no Japão ocupado. Alinhada aos estudos sobre circulação do conhecimento, esta análise se volta para os trabalhos produzidos por Hall sobre a educação brasileira e japonesa, argumentando que sua experiência no Brasil foi importante para sua atuação como intelectual a serviço do governo estadunidense.

Em 1940, Robert King Hall (1912-1981)^1^ chegou ao Brasil para estudar as reformas do ensino secundário realizadas pelo governo de Getúlio Vargas (1930-1945). Cursando doutorado em educação comparada pela Universidade de Michigan (UM), ele investigava o controle federal do ensino secundário em três repúblicas latino-americanas: Argentina, Brasil e Chile. A viagem ao Brasil ocorreu graças a uma bolsa de estudos, com duração de seis meses, concedida pelo Instituto Brasil-Estados Unidos (Ibeu) por meio do Brazilian Fellowship Program (BFP), convênio firmado entre o instituto e a UM (Kropf, maio-ago. 2020). O acordo atendia aos ideais propagados pela Política da Boa Vizinhança, cujo objetivo era promover boas relações entre EUA e América Latina por meio de acordos econômicos e políticos e de intercâmbios científicos e culturais (Ninkovich, 1981; Pike, 1995; Sadlier, 2012; Smith, 2017; Valim, Mauad, 2023).

No Brasil, Hall percorreu o país visitando escolas, realizando palestras e estabelecendo contatos com educadores como Lourenço Filho, Paschoal Lemme e Anísio Teixeira. Contou, ainda, com a colaboração do Instituto Nacional de Estudos Pedagógicos (Inep) no acesso a documentos e informações sobre a educação brasileira. Além do ensino secundário, chamou a atenção do pesquisador estadunidense a questão dos imigrantes que, até o início dos anos 1940, viviam isolados, preservando a língua, a educação e a cultura de seus países de origem. Interessado no assunto, Hall visitou os estados de Santa Catarina e São Paulo para observar as ações do Estado Novo (1937-1945), no sentido de nacionalizar os estrangeiros. Em São Paulo, acompanhou o fechamento de escolas clandestinas, entrando em contato com livros e métodos didáticos japoneses. Preocupado com a presença de súditos do Eixo (Alemanha, Itália e Japão) no Brasil e o perigo que esses grupos poderiam representar para o futuro das relações interamericanas, Hall enviou um relatório para a embaixada estadunidense, expondo suas observações sobre as colônias alemãs e japonesas.

Ao retornar aos EUA, Hall mobilizou os conhecimentos adquiridos no Brasil para servir aos interesses geopolíticos de seu país. Inicialmente, atuou, em 1943, no National Japanese American Student Relocation Council (NJASRC), cuja função era ajudar universitários nipo-americanos internados em campos de concentração a retomarem seus estudos (Niiya, 1993). Em seguida, coordenou uma equipe de tradutores militares responsável pela tradução de livros didáticos japoneses. Por fim, em 1945, quando o Japão foi derrotado e ocupado pelas Forças Aliadas, Hall serviu ao governo estadunidense, trabalhando nas reformas educacionais realizadas no país asiático.

Este artigo – fruto da minha tese de doutorado, defendida em 2024, no Programa de Pós-graduação em História das Ciências e da Saúde da Casa de Oswaldo Cruz/Fiocruz – examina a relação entre as observações de Robert King Hall sobre a nacionalização dos imigrantes pelo Estado Novo e sua atuação no Japão no pós-Segunda Guerra. Para tanto, utilizo como fontes os trabalhos produzidos por Hall sobre a educação brasileira e japonesa – compostos pela sua tese, por seus artigos, livros e relatórios –, assim como decretos e matérias de jornais que divulgaram sua passagem pelo Brasil. A análise alinha-se aos estudos sobre circulação transnacional de conhecimentos, pessoas e práticas, procurando contribuir com abordagens que evidenciam a não linearidade de tais fluxos, ressaltando as modificações e construções que ocorrem mediante os encontros e disputas entre os vários atores que neles transitam (Secord, dez. 2004; Raj, 2007; Fan, 2012; Raposo et al. 2014).

Ao investigar como Hall mobilizou os conhecimentos adquiridos no Brasil para colaborar com a política externa estadunidense, argumento que sua experiência no país serviu de “laboratório” para a formulação de suas propostas educacionais. Essa viagem permitiu que ele fosse convocado a atuar no Japão, contribuindo, decisivamente, para o seu reconhecimento como “especialista” em educação, levando-o a atuar, durante a Guerra Fria, como consultor técnico em países como Brasil, Arábia Saudita e Irã.

O artigo se encontra dividido em quatro partes. Na primeira, trato das negociações para a viagem de Hall ao Brasil em 1940, seus interesses de estudo, as conexões estabelecidas com o campo educacional brasileiro e o papel da Política da Boa Vizinhança na promoção dos intercâmbios entre Brasil e EUA. Em seguida, apresento os apontamentos de Hall sobre o processo de nacionalização dos imigrantes, destacando a importância conferida ao tema no contexto de guerra, quando os EUA procuravam combater a influência do Eixo na América Latina, tornando o Brasil seu principal aliado no continente. Na terceira parte, exploro a atuação de Hall no Japão, discutindo suas propostas para reformar a educação japonesa, relacionando-as com suas investigações no Brasil. Por fim, indico os caminhos percorridos por Hall após voltar aos EUA, incluindo novas viagens ao Brasil e a regiões de interesse do governo estadunidense durante a Guerra Fria.

## Política da Boa Vizinhança, intercâmbios educacionais e a viagem de Hall ao Brasil

Em 1936, na Conferência Interamericana para Manutenção da Paz, realizada em Buenos Aires, o presidente Roosevelt expressou, na Convention for the Promotion of Inter-American Cultural Relations, o interesse do governo estadunidense em estabelecer cooperação cultural com os países latino-americanos, comprometendo-se a promover uma política oficial de financiamento para a área. Tal medida estava em consonância com a Política da Boa Vizinhança, cujo objetivo era fortalecer os laços entre EUA e América Latina por meio de acordos econômicos e políticos e de intercâmbios científicos e culturais (Ninkovich, 1981; Sadlier, 2012; Smith, 2017).

A adoção da Boa Vizinhança representava uma mudança na política externa dos EUA em relação à América Latina, marcada durante muito tempo pela força do *big stick*. Essa transformação resultava de uma série de fatores, a começar pelo crescimento do antiamericanismo entre os latino-americanos, especialmente na América Central e no Caribe, palco de diversas intervenções militares (Schoultz, 2000). O contexto internacional, marcado pela ascensão do fascismo e do nazismo, parecia propício a um novo conflito mundial. No caso da Alemanha, causava apreensão aos EUA o crescimento das relações comerciais entre o regime de Hitler e países da região, a exemplo do Brasil. A presença de imigrantes alemães, italianos e japoneses, mantendo fortes laços com seus países de origem, e a admiração dos militares latino-americanos pela máquina de guerra alemã eram também motivos de preocupação para o governo estadunidense (Moura, 1980).

Os EUA decidiram, então, investir no estabelecimento de uma diplomacia cultural pública, contando com a colaboração de instituições privadas que, desde o início do século XX, já atuavam nessa área (Ninkovich, 1981; Faria, 2002).^2^ Em 1938, foi criada, no Departamento de Estado, a Divisão de Relações Culturais, para viabilizar as trocas com os latino-americanos por meio da promoção de intercâmbios patrocinados com recursos do governo estadunidense. Dois anos depois, o Office of the Coordinator of Inter-American Affairs, comandado pelo empresário Nelson Rockefeller, passou a organizar acordos econômicos e intercâmbios educacionais e culturais, investindo, sobretudo, no uso do rádio, do cinema, da imprensa e da propaganda (Tota, 2000; Graham, 2015; Valim, Mauad, 2023).

Vale ressaltar que muitos países consideravam as trocas culturais importantes para a sua política externa e, por isso, incentivavam a criação de institutos binacionais e intercâmbios científicos (Lessa, 1998; Sá et al., 2009; Silva, 2010). No caso do Brasil, o governo investiu em mais aproximação com a América Latina, por meio da criação de missões culturais em vários países da região (Suppo, Lessa, 2012). Nesse sentido, os institutos binacionais assumiam papel de destaque como espaços de divulgação da cultura, ampliando os contatos entre as nações. Foi o caso do Instituto Brasil-Estados Unidos, que, criado em 1937, não apenas oferecia cursos de inglês, também atuava na promoção das relações entre os dois países por meio da organização de palestras e exposições, na recepção de intelectuais e artistas e nos intercâmbios educacionais.

Por iniciativa do Ibeu, em 1938, foi firmado um convênio entre o instituto e a UM, o Brazilian Fellowship Program (BFP), com o objetivo de promover o intercâmbio educacional entre os dois países. O BFP estava em consonância com a Política da Boa Vizinhança e atendia aos interesses da UM de atrair estudantes estrangeiros para o Meio-Oeste estadunidense, ao mesmo tempo que colocava o Ibeu nas rotas de circulação estabelecidas pela diplomacia cultural interamericana. Pautado no ideal de reciprocidade, o BFP estabelecia que o Ibeu forneceria três bolsas para estudantes estadunidenses e a UM concederia a mesma quantidade de *fellowship* para brasileiros (Kropf, 2020).

Robert King Hall, que cursava o doutorado em educação comparada pela UM, encontrou nessa iniciativa a oportunidade ideal para expandir suas pesquisas sobre a América Latina. Seu contato com a educação latino-americana era anterior ao BFP, visto que ele já havia investigado o ensino secundário argentino durante o mestrado na Universidade de Chicago. Naquele período, ele realizou sua primeira incursão pela região, em 1935, visitando nove países (Panamá, Colômbia, Equador, Peru, Bolívia, Chile, Argentina, Paraguai e Brasil) durante três meses. As impressões dessa viagem foram expostas em artigo publicado no *Bulletin of the Pan-American Union*, no qual Hall (jul. 1936, p.549) defendia que “dólar por dólar, você pode ir mais longe, ver mais e viajar melhor na América do Sul do que na Europa”.^3^ Seu objetivo era convencer os compatriotas a conhecer as belezas e a riqueza cultural latino-americana, estabelecendo uma relação de “amizade” com os vizinhos.

O Brasil, que até então não era objeto de suas investigações, foi inserido na sua rota de circulação após Hall ser convidado a integrar o trio de estudantes que viriam ao país com bolsa fornecida pelo Ibeu. Entre junho e outubro de 1940, o pesquisador visitou os seguintes estados: Amazonas, Bahia, Ceará, Espírito Santo, Goiás, Maranhão, Minas Gerais, Pará, Paraná, Pernambuco, Rio de Janeiro, Rio Grande do Sul, Santa Catarina e São Paulo. Durante as pesquisas, realizou entrevistas e obteve acesso a informações e à ajuda logística de governadores e educadores, especialmente do diretor do Inep, Lourenço Filho, que, além do acesso a fontes, ajudou Hall a traçar seu roteiro de viagem. Inclusive, o diretor do Inep estivera nos EUA, em 1935, para realizar estudos no Teachers College da Universidade de Columbia, onde Hall viria a atuar a partir de 1947. Além dele, outros brasileiros, como Noemy Rudolfer, Anísio Teixeira e Isaías Alves, também tiveram contato com a educação estadunidense por meio de viagens e estudos realizados com bolsas fornecidas por instituições filantrópicas ou patrocinados pelas secretarias de educação estaduais onde atuaram, na década de 1920, realizando importantes reformas (Rocha, 2020).

No Inep, Hall foi auxiliado por Paschoal Lemme, diretor da seção de documentação do instituto que também participou, em 1939, do intercâmbio entre o Ibeu e a UM. Foi Lemme quem apresentou o pesquisador a Anísio Teixeira, que, naquele período, se encontrava na Bahia e recebeu Hall para uma conversa (Lemme, 2004). De lá, Hall seguiu para Pernambuco, onde foi recepcionado por Gilberto Freyre em Recife (No Recife..., 3 set. 1940). Os dois se conheceram nos EUA, em 1939, quando, no Institute of Latin-American Studies, lecionaram um conjunto de cursos e seminários realizados durante a *summer session* da Universidade de Michigan (Hall, abr. 1939). Nesse percurso, Hall proferiu palestras na Faculdade de Filosofia do Paraná, na secretaria de educação da Bahia, no Ibeu e na Universidade do Brasil (Conferências, 19 jun. 1940).

Buscando estabelecer a relação entre educação e regimes políticos, a tese de Hall, intitulada *Federal control of secondary education in the ABC Republics*, tinha por objetivo determinar se as reformas educacionais realizadas na Argentina, no Brasil e no Chile eram “um instrumento para providenciar oportunidades educacionais para a juventude” ou estariam “produzindo uma ordem social que consistirá numa ameaça à democracia” (Hall, 1941a, p.4). A preocupação de Hall justificava-se pelo contexto da guerra e pela ameaça que a Alemanha nazista representava para a posição dos EUA no continente. Existia um temor de que os países latino-americanos se aliassem ao Eixo. No caso do Brasil, Getúlio Vargas, que governava por meio da ditadura do Estado Novo, havia adotado uma posição de “equidistância pragmática” em relação ao conflito, negociando com EUA e Alemanha, o que causava forte desconfiança por parte do governo estadunidense (Moura, 1980).

Tomando a Alemanha como laboratório de um grande experimento totalitário, Hall (1941a, p.2) argumentava que a ascensão de governos autoritários na Europa havia deixado evidente que “o sistema educacional, em seu mais amplo sentido, era destinado a desempenhar um papel extremamente importante na nova ordem mundial” (p.1). Nesses regimes, a educação estava voltada para a formação de soldados, cidadãos obedientes a um líder. Por isso, interessava-lhe investigar as reformas do ensino secundário, tendo em vista que essa modalidade de ensino tinha por função a formação da juventude e da força de trabalho. Para Hall, o modelo de educação adotado por um país estava diretamente ligado ao seu sistema político. Portanto, um ensino demasiadamente nacionalista e centralizador poderia ser um indício de virada rumo ao autoritarismo. No caso da América Latina, isso representaria uma ameaça direta à hegemonia dos EUA no continente.

Após seis meses de viagem, no entanto, Hall chegou à conclusão de que, embora o Brasil não fosse uma democracia, o governo de Vargas não representava uma ameaça à segurança hemisférica. O ensino secundário, criticado pelo pesquisador por ser “enciclopédico demais”, “elitista” e “inadequado para o tamanho do território brasileiro e pelas desigualdades regionais”, não era o principal problema da educação brasileira (Hall, 1941a). Afinal, a maior parte da população era analfabeta e nem sequer chegava ao ensino secundário, portanto, o problema real, concluiu, estava nas altas taxas de analfabetismo e na concentração de imigrantes de países do Eixo que se recusavam a se integrar ao Brasil.

Foi justamente a questão dos imigrantes que chamou a atenção de Hall, levando-o a visitar os estados de Santa Catarina e São Paulo para acompanhar de perto as ações de nacionalização realizadas pelo Estado Novo. Suas observações e impressões foram, posteriormente, sistematizadas num relatório enviado à embaixada estadunidense e apresentadas em artigos publicados nos EUA, tornando-o reconhecido como um “especialista” em América Latina, mais especificamente na organização das colônias alemãs e japonesas no Brasil (Robert..., 1942).

## A nacionalização dos imigrantes e o combate à influência do Eixo no Brasil

A partir dos anos 1930, o projeto nacionalizador de Getúlio Vargas colocou em evidência a questão do isolamento dos imigrantes. Além das diferenças linguísticas e culturais, quando chegaram ao Brasil, alemães, japoneses e italianos haviam se deparado com a falta de escolas públicas para a instrução de seus filhos. Vindos de países de forte tradição escolar, esses imigrantes procuraram construir estruturas próprias para a oferta de ensino nas regiões onde se instalaram (Kreutz, 2000). A organização dessas colônias se tornou, então, um entrave para o Estado Novo, que buscava imprimir um caráter nacional ao ensino por meio da padronização de currículos, da sistematização de conteúdos e da supressão de elementos estrangeiros.

Para além de questões internas, a iminência de um conflito mundial e a presença de imigrantes de origem alemã, japonesa e italiana não assimilados causava apreensão não só ao governo brasileiro, como também aos EUA, que temiam um avanço nazista no continente. Para resolver o problema, Vargas iniciou um programa de nacionalização por meio de uma série de decretos-lei promulgados entre 1938 e 1942. Entre as medidas adotadas estavam a proibição do uso de idiomas estrangeiros em estabelecimentos comerciais; a limitação de entrada de imigrantes; a obrigatoriedade do ensino em língua portuguesa e do ensino de história e geografia do Brasil em todas as escolas (Brasil, 30 dez. 1938). A partir de 1939, com a criação da Comissão Nacional de Ensino Primário, o governo federal passou a construir, em parcerias com os estados, escolas primárias que deveriam substituir as de imigrantes.

Preocupado com uma possível influência nazista, o governo tomou medidas para controlar a produção, a importação e o uso de livros didáticos no país. Criou, ainda, a Comissão Nacional do Livro Didático, responsável, entre outras coisas, por examinar as obras e autorizar ou não o seu uso; estimular a produção nacional; indicar livros didáticos estrangeiros para serem traduzidos; promover exposições nacionais daqueles que foram autorizados para utilização (Brasil, 30 dez. 1938). Temia-se que os livros estrangeiros pudessem alimentar o separatismo e o desprezo pelas instituições nacionais.

Nesse sentido, atuaram conjuntamente o Ministério da Educação e Saúde Pública e o Ministério da Guerra. Enquanto o primeiro se responsabilizava pela organização do ensino público, o segundo deveria investigar e coibir quaisquer reações ao processo nacionalizador, garantindo a segurança nacional, fechando escolas clandestinas e combatendo aliados do Eixo. Esse processo foi marcado por prisões, perseguições políticas e arbitrariedades cometidas sob a justificativa da guerra (Seyferth, 1999). Ao mesmo tempo, vários grupos procuraram, clandestinamente, conservar as escolas étnicas.

O governo estadunidense, atento ao problema dos imigrantes, chegou a realizar investigações para dimensionar o perigo que esses grupos representavam para o Brasil e para o continente (Tota, 2000). Portanto, não é estranho que Hall tenha enviado, em agosto de 1940, um relatório para a embaixada estadunidense apresentando o que viu ao visitar colônias alemãs e japonesas durante sua passagem pelo país. Esse documento é uma forte evidência de que suas pesquisas na América Latina tinham não somente um sentido acadêmico, mas também uma dimensão claramente política, diretamente associada aos interesses de seu país na região.

Na tese, o tema dos estrangeiros recebeu pouca atenção, visto que sua pesquisa focalizava as reformas do ensino secundário. Já no relatório, Hall revelava um olhar atento para o problema da nacionalização dos alemães em Santa Catariana e dos japoneses em São Paulo. Alguns posicionamentos apresentados nesse documento seriam, posteriormente, destacados nos artigos que publicou após retornar aos EUA. As observações foram importantes não só para compreender como viviam e se organizavam os colonos dos países do Eixo, mas também para entender como o governo Vargas combatia essa influência e até que ponto ela era uma ameaça para a segurança do continente.

Essa questão aparece no artigo “Foreign colonies of Brazil: a North American view”, no qual Hall (1941b) se dispõe a apresentar uma visão “neutra” do conflito entre brasileiros e estrangeiros no processo de nacionalização. Sua análise começava pelo fechamento das escolas, responsável, a seu ver, por “uma grande quantidade de atritos e problemas desnecessários” (p.13). O pesquisador apontava para a violência do governo brasileiro para com os imigrantes em São Paulo, Santa Catarina e Paraná. Por outro lado, afirmava que “os brasileiros estão corretos quando dizem que as escolas foram inundadas com propaganda nazista” (p.13). Hall destacava que em muitos estabelecimentos clandestinos foram encontrados materiais de propaganda nazista que indicavam “a necessidade do *Lebensraum* [espaço vital] para o povo alemão, a superioridade do exército e a localização de organizações nazistas em várias partes do mundo” (p.13). Entretanto, ele ressaltava que nem todos os alemães eram, necessariamente, seguidores do nazismo ou partilhavam dessas teorias, sendo injustas as ações violentas praticadas pelo governo indiscriminadamente. Suas críticas não eram voltadas para a política de nacionalização em si, mas para as truculências cometidas durante o processo.

Hall reconhecia que o Brasil vivia sob uma ditadura caracterizada por censura e perseguição aos opositores do regime. Esse ponto fica mais evidente no relatório, quando o pesquisador tece críticas diretas ao regime varguista. Se nos artigos ele assumia uma posição conciliatória, no documento ele criticava abertamente o trabalho realizado pelos inspetores brasileiros, apontados como “tecnicamente ignorantes”. Segundo Hall (14 ago. 1940, p.3), uma das razões que explicavam o conflito entre alemães e brasileiros era o fato de que muitos dos envolvidos na condução do processo de nacionalização eram nitidamente negroides do Norte do Brasil, exercendo atividades sobre alemães brancos e puros com muita consciência racial. Em outro trecho, Hall ressaltava a dificuldade enfrentada pelo governo brasileiro “de convencer os alemães a mudar uma cultura forte, reconhecidamente superior, que existia há três gerações” (p.4). Percebe-se que, ao se dirigir aos representantes do governo estadunidense, Hall se mostrava à vontade para expressar comentários racistas, revelando uma visão preconceituosa em relação aos brasileiros, apontados por ele como inferiores diante de alemães com uma cultura “superior”.

Outro ponto destacado por Hall (1941a, p.180) foi o controle dos livros estrangeiros em circulação no país, ressaltando a severidade com que o governo estava lidando com a questão. Segundo ele, atividades como essas eram realizadas sem critérios seletivos e, em muitos casos, resultavam no confisco de obras estrangeiras que não tinham nenhum tipo de ligação com ideologias como o comunismo e o nazismo. A censura também prejudicava a circulação de livros estadunidenses, o que, para Hall, não fazia nenhum sentido, levando em consideração as relações de amizade entre Brasil e EUA.

Os prejuízos não se resumiam aos livros, mas também à propagação das ideias, da língua e da cultura estadunidense entre os brasileiros. Em outro artigo, “English teaching in Argentina and Brazil”, Hall (1942) tratou do ensino da língua inglesa na Argentina e no Brasil. No caso deste último, seu objetivo foi demonstrar como a política repressiva estadonovista, direcionada aos estrangeiros, limitava a propagação do idioma no país. Segundo Hall, os brasileiros mostravam-se muito interessados em conhecer a cultura estadunidense (p.79). O empecilho estava posto apenas no plano político, pois, com a descoberta de estabelecimentos clandestinos, aumentara o antagonismo do governo brasileiro em relação às línguas estrangeiras.

Para ele, essas medidas eram injustas, uma vez que as relações entre Brasil e EUA estavam pautadas na “sinceridade das intenções”. Como bons vizinhos e aliados na luta contra o Eixo, os estadunidenses não deveriam receber o mesmo tratamento que alemães e japoneses. Hall (1942, p.81) destacava, ainda, a crescente “popularização do inglês e da cultura estadunidense” entre os brasileiros como mais uma justificativa da proximidade entre as duas nações.

Em relação aos japoneses, Hall (1941b, p.18) atribuiu aos nipônicos uma forte relutância a serem nacionalizados, o que se justificaria pelas marcantes diferenças culturais em relação aos brasileiros. Esses posicionamentos ficam mais claros no relatório, no qual ele apontava existir na relação entre japoneses e brasileiros um “choque de raças”, visto que os dois lados apresentavam visões muito negativas um do outro. Se, para os brasileiros, os japoneses “são sujos” e “indignos de confiança”, para os nipônicos, a educação brasileira era “medíocre” e as autoridades “esperavam suborno” (Hall, 14 ago. 1940, p.8). Na avaliação de Hall, essas diferenças tornavam muito difícil a assimilação dos japoneses, defendendo ser mais pertinente “acabar com toda nova imigração” (p.12).

A crença na incapacidade de assimilação dos japoneses era partilhada por muitos intelectuais e políticos brasileiros, que já discutiam essa questão muito antes da guerra. A imprensa e os discursos científicos contribuíram para disseminar essa visão, classificando os europeus como imigrantes ideais para o país, enquanto africanos e asiáticos seriam completamente estranhos à nossa cultura (Shizuno, 2010; Morais, 2011).

Chegados ao Brasil a partir de 1908 para trabalhar nas lavouras de café, os japoneses haviam se espalhado pelos interiores de São Paulo e do Paraná. Nessas regiões, desenvolveram colônias onde buscavam preservar o *yamatodamashi*, valores e padrões culturais japoneses (Cytrynowicz, Cytrynowicz, 2017). O preconceito, aliado às diferenças linguísticas e culturais, fazia com que se mantivessem isolados, mantendo-se como japoneses residentes no Brasil (Tanno, 2008). Com a guerra, os preconceitos deram vazão a violências e restrições. Em São Paulo, o governo estadual acompanhou os decretos federais, promovendo despejos, vigilância e controle dos japoneses. O fechamento das escolas não foi aceito pelos colonos, que resistiram e mantiveram estabelecimentos clandestinos. Mais do que instrução, os japoneses viam nelas o local de aprendizagem dos valores essenciais do *yamatodamashi* (Morais, 2011).

A manutenção desses valores e a construção de colônias fechadas e bastante organizadas econômica, social e culturalmente, associadas às exigências educacionais dos japoneses, foram apontadas por Hall como elementos que impediam a nacionalização desse grupo. No relatório, o pesquisador destacava que, mesmo matriculando seus filhos em escolas brasileiras, os japoneses mantinham-nos em estabelecimentos clandestinos que funcionavam à noite ou de madrugada. Segundo seu relato, ele “estava presente quando uma delas foi invadida e 27 alunos e dois professores foram levados às 23:30” (Hall, 14 ago. 1940, p.8).

A partir de uma análise do material apreendido pela polícia, Hall (14 ago. 1940, p.7) identificou o uso de materiais didáticos que continham “mapas e gráficos de propaganda”, demonstrando a “força militar de vários países, bandeiras do Japão, fotos do imperador e de homens que morreram como bomba humana”. Quanto aos livros didáticos apreendidos, o pesquisador destacava a presença de elementos como fotos de “aviões, metralhadoras e bandeiras do Japão, Alemanha e Itália”. Anexo ao relatório, encontravam-se exemplares de livros apreendidos durante operação policial na cidade de Marília, São Paulo. É possível identificar vários dos elementos destacados por Hall: escritos em japonês, apresentavam inúmeras fotos de figuras militares e bandeiras. O conteúdo era ensinado a partir de elementos da história e da cultura japonesa, tendo em vista exaltar o país.

Essas observações foram úteis, posteriormente, para o governo estadunidense, que, no pós-guerra, iniciou o processo de ocupação do Japão. Nesse sentido, os conhecimentos adquiridos por Hall no Brasil foram decisivos não só para o seu reconhecimento como “especialista”, mas também para sua atuação nas reformas do sistema educacional japonês. Sua pesquisa, inicialmente voltada para o ensino secundário, ganhou novas dimensões a partir da visita que realizou a colônias japonesas. Isso só foi possível porque Hall conseguiu estabelecer contatos com educadores e instituições brasileiras, o que lhe permitiu acompanhar de perto o processo de nacionalização. Esse caso demonstra como, nos processos de circulação, ao mesmo tempo que podemos identificar o pertencimento do viajante a uma dada configuração social, baliza que dá sentido ao que ele coleta, notamos também a incorporação de novos interesses a partir dos encontros que realiza ao longo do trajeto (Raposo et al., 2014).

## Conhecimentos em circulação: a atuação de Hall no Japão (1945-1946)

Depois do ataque a Pearl Harbor, em dezembro de 1941, os conhecimentos adquiridos por Hall sobre as colônias japonesas no Brasil se tornaram úteis para o esforço de guerra, tendo em vista que, nos EUA, muito pouco se conhecia sobre a cultura e os valores japoneses (Benedict, 2014). Em 1943, o pesquisador estadunidense foi designado para atuar no NJASRC, criado com o objetivo de ajudar os estudantes nipo-americanos internados em campos de concentração a retornar à universidade (Niiya, 1993). Foi nesse período que Hall se alistou na Marinha e passou a frequentar a Naval School of Military Government and Administration na Universidade de Columbia, escola voltada para o treinamento de oficiais que atuariam no Pacífico (Wallace, 1944). Já em 1945, ele seguiu para Monterey, Califórnia, onde trabalhou como chefe da Seção de Educação do Planning Staff for the Occupation of Japan at the Civil Affairs Staging Area, passando a liderar um grupo de militares responsáveis pela tradução dos livros didáticos japoneses (Hall, 1949a).

Acabada a guerra, o Japão foi ocupado pelas Forças Aliadas, sob a liderança do general estadunidense Douglas MacArthur, o *supreme commander for the Allied Powers* (Scap). Mais uma vez, a trajetória de Robert King Hall seguiria os caminhos da política externa estadunidense, levando-o a atuar nas reformas do sistema educacional nipônico. No Japão, ele foi nomeado *chief of the Education Sub-Section of the Civil Information and Education Section of the Civil Information and Education Division* (CI&E), agência encarregada de auxiliar o Ministério da Educação com a reforma (Hall, 1949a, p.4). Entre as atividades do CI&E estava a supervisão dos currículos escolares e a avaliação dos livros didáticos (Nishi, 1982).

No pré-guerra, a educação japonesa estava pautada pelos princípios confucionistas e pela promoção do nacionalismo e do ultranacionalismo. Portanto, se os EUA quisessem estabelecer a desmilitarização da sociedade japonesa, a reforma educacional era indispensável para atingir tal objetivo (Mello, 2006). Para eliminar o nacionalismo e o militarismo, o Scap extinguiu os cursos de *shushin* (disciplina de moral e cívica ministrada nos cursos primários) e investiu na liberalização do ensino, com a demissão de funcionários militares das escolas e a exclusão de conteúdos militaristas presentes nos livros didáticos (Mello, 2006).

Na avaliação de Hall (1949b, p.5), “um Japão pacífico é a chave para um mundo em paz, e a reeducação das pessoas é a chave para um Japão pacífico”. A questão era como fazer aquele país, com cultura, religião e valores tão distintos dos estadunidenses, adotar a democracia como sistema político. Para ele, a resposta estava no sistema educacional que seria adotado pelos japoneses no pós-guerra e, por isso, compreender como a educação nipônica funcionava era essencial para ajudar na reconstrução do sistema educacional japonês e na reforma da estrutura política japonesa (Hall, 1949b, p.8).

Ao se debruçar sobre a história japonesa, Hall (1949b, p.33) se mostrou impressionado com o programa educacional nipônico, marcado pela educação em massa e por altos investimentos em recursos humanos. Reconhecia também a capacidade dos japoneses de inserir diferentes modelos educacionais, misturando-os e adaptando-os aos seus valores e interesses. Ao mesmo tempo, apontava para os perigos e as consequências da centralização da educação nas mãos do *Monbusho* (Ministério da Educação).

Cabia ao *Monbusho* “o controle sobre a seleção, o treinamento e a supervisão de professores, o conteúdo dos currículos, a preparação de livros didáticos e a censura a livros comerciais” (Hall, 1949b, p.33). Esse órgão era igualmente responsável por manter a unidade nacional por meio “do ensino de conceitos morais e éticos” e dos cursos de *shushin*, promovendo “a vigilância e controle de pensamento”. As críticas de Hall ao sistema educacional e ao Ministério da Educação japoneses recaíram sobre este último ponto, marcado pela transformação dos princípios éticos em mecanismos de controle social.

Em 1937, o *Monbusho* publicou o documento intitulado *Kokutai no Hongi*, responsável por orientar a organização dos cursos de *shushin* e a produção dos livros didáticos. O primeiro contato de Hall com esse material ocorreu no Brasil, em agosto de 1940, quando ele acompanhou uma operação policial numa escola clandestina na cidade de Marília. O que chamou sua atenção foi a presença do *kokutai* como espécie de “manual” para a educação dos colonos japoneses (Hall, 1949a, p.25-26).

Segundo Hall (1949b, p.71), o *kokutai* teve uma influência determinante na educação japonesa, com uma grande tiragem e circulação, servindo de leitura obrigatória para professores e alunos de ensino médio e regendo o ensino de ética das escolas. A importância atribuída ao *kokutai* pode ser medida pelo fato de que a primeira ação do Scap foi a de suspender os cursos de *shushin* nas escolas japonesas.

Em *Education for a new Japan*, Hall (1949c) discutiu as primeiras ações do Scap no sentido de reformar a educação japonesa. No livro, Hall expressava sua avaliação: “O sistema educacional japonês continua tão potencialmente perigoso quanto era antes de Pearl Harbor” (p.202). A principal razão seria o ponto de maior controvérsia entre Hall e as forças de ocupação: a reforma do sistema de escrita japonês. Em um memorando intitulado “The exclusive use of katakana as official written language”, enviado, em 23 de junho de 1945, para a Divisão de Assuntos Civis do Departamento de Guerra, Hall recomendava a proibição do uso de *kanji*, tornando obrigatório o uso exclusivo do *katakana* (Unger, 1996).

Antes de mais nada, é preciso compreender como funciona o sistema de escrita japonês, que consiste em três tipos de alfabeto: *hiragana*, *katakana* e os caracteres chineses, chamados de *kanji*. Cada um deles tem diferentes formas de uso, propósitos e características. O *hiragana* é usado para palavras nativas japonesas e elementos gramaticais, sendo a base do idioma. Já o *katakana* é usado para palavras e nomes que não têm origem japonesa, empréstimos linguísticos e onomatopeias. E o *kanji*, considerado o mais difícil de dominar, tem origem chinesa, podendo ultrapassar mais de dez mil letras, sendo que cada *kanji* pode ter mais de um significado. É possível também representar a forma escrita japonesa utilizando caracteres do alfabeto latino por meio do processo de romanização, que recebe a denominação de *romaji*, frequentemente utilizada pelos estrangeiros que não conhecem as formas de escrita japonesa (Ota et al., 2001).

A proposta de Hall seria excluir o *kanji,* de origem chinesa, e adotar apenas o *katakana,* empregado para palavras e nomes estrangeiros e, portanto, menos usado pelos japoneses. Segundo ele, a dificuldade excessiva da escrita japonesa era um obstáculo ao desenvolvimento da democracia, daí a necessidade de uma reforma linguística. Na sua avaliação, “a forma escrita do japonês é simplesmente muito ineficiente para ser continuada se o Japão quiser competir numa base aproximada de igualdade com as nações ocidentais” (Hall, 1949c, p.300). O pesquisador argumentava que os próprios japoneses acusaram a forma escrita da língua de constituir “uma grande barreira à reconstrução do sistema educativo e à democratização do país” (p.312). As dificuldades apresentadas na aprendizagem da escrita japonesa, segundo Hall, revelavam uma espécie de elitismo intelectual, visto que, em geral, apenas as pessoas mais abastadas conseguiam prosseguir os estudos, dedicando tempo para dominar a sua forma escrita (p.312).

Ao chegar ao Japão, Hall descobriu que já existiam propostas, elaboradas por linguistas japoneses, para reformar o sistema de escrita. Desde 1909, quando foi criado o Primeiro Conselho Nacional de Pesquisa de Línguas, estudiosos tentavam limitar o número de caracteres chineses utilizados. Entretanto, o ultranacionalismo que vigorou no período pré-guerra impediu que tais movimentos crescessem no país, sendo reativados com a ocupação. Segundo Gottlieb (1998, p.71), muitos especialistas utilizaram “o novo conceito de democracia importado pelas forças de ocupação para fornecer um argumento poderoso a favor da racionalização da forma como o japonês era escrito”. Os japoneses defensores dessas mudanças se aliaram a alguns estadunidenses que, como Hall, viam na complexidade da escrita um entrave para a democratização do país (Krumrey, 1993). Nesse grupo estavam desde reformadores radicais, que defendiam a completa eliminação dos *kanjis*, até os que assumiam posições mais moderadas e propunham uma limitação no número de caracteres (Gottlieb, 1998).

Ao entrar em contato com especialistas japoneses, Hall se apropriou de um debate local para pensar maneiras de facilitar o que ele considerava ser a transformação do Japão numa democracia alinhada aos ideais estadunidenses. Ao mesmo tempo, estudiosos japoneses utilizaram-se da retórica da democracia para colocar em pauta propostas de reforma há muito tempo defendidas (Gottlieb, 1998). Segundo Tsuchimochi (1993), ao chegar ao Japão e entrar em contato com os debates locais, Hall mudou sua proposta, sugerindo o uso do *romaji* em vez da proposta original que previa o uso de *katakana*. Junto com Jiro Aramitsu, chefe do Departamento de Livros Didáticos, Hall propôs a romanização dos livros didáticos japoneses, justificando que eles tornariam “mais fácil para os estrangeiros lerem em japonês” e “mais fácil para o povo ler leis e jornais e tornar-se mais alfabetizado” (Tsuchimochi, 1993, p.27).

Essa situação aponta para a multiplicidade de agentes e interesses envolvidos nos processos transnacionais de circulação de conhecimentos, mostrando como, em muitos casos, os agentes locais não só estavam conscientes como se beneficiavam da presença estrangeira para mobilizar agendas próprias. Conforme assinala Kapil Raj (2007), a circulação é um movimento de encontros, marcado por interações entre atores e grupos heterogêneos, promovendo a formação de redes e a produção de novos conhecimentos no âmbito dos próprios circuitos da circulação.

Ao propor a reforma, Hall estava defendendo os interesses estadunidenses, visando tornar o Japão, antes inimigo, em seu principal aliado no continente asiático. Para ele, o sistema de escrita japonês era “uma barreira à compreensão e amizade internacionais”, visto que “a dificuldade excessiva do sistema de escrita tornou a cultura japonesa relativamente inacessível aos ocidentais” (Hall, 1949c, p.312-313). Isso dificultava as próprias ações do Scap, visto que “as massas populares não foram devidamente alcançadas pelos serviços de informação das autoridades de ocupação” (p.313). Além dos documentos oficiais estarem escritos, originalmente, em inglês, Hall apontava que a tradução para o japonês “foi considerada difícil, senão impossível” (p.313). Ou seja, ele estava propondo uma reforma que facilitaria o processo de ocupação do Japão por meio de mudanças estruturais não apenas no seu sistema político, mas na língua e na cultura nipônicas.

As propostas de Hall geraram inúmeros debates que envolveram membros da missão educacional estadunidense, especialistas japoneses e funcionários do Scap e do Departamento de Estado. Entretanto, assim como a proposta para proibição do uso de *kanjis*, a sugestão de romanização dos livros didáticos não foi autorizada. Depois de conversar com o ministro da Educação, Tamon Moeda, o chefe da Divisão de Educação do CI&E, Harold G. Henderson, tomou a decisão, o que gerou atritos entre Hall e membros da divisão. Posteriormente, o general MacArthur realizou algumas mudanças no órgão, e Hall deixou a divisão de livros, passando para a divisão de planejamento, sendo, por fim, desligado de funções relativas ao Ministério da Educação (Tsuchimochi, 1993, p.28).

## Guerra Fria e novas rotas de circulação

No final de 1946, Robert King Hall retornou aos EUA. No ano seguinte, passou a ocupar a cadeira de educação comparada do Teachers College da Universidade de Columbia. Se os conhecimentos adquiridos durante a viagem ao Brasil foram cruciais para sua inserção na missão educacional no Japão, sua experiência no país asiático terminou por consolidar seu nome como uma referência em estudos latino-americanos e japoneses. As pesquisas desenvolvidas por ele e os contatos estabelecidos com o campo educacional brasileiro foram fundamentais para projetá-lo como especialista em educação comparada, cuja trajetória estaria indissociavelmente ligada aos interesses geopolíticos estadunidenses.

Em 1948, ele retornou ao Brasil para “visitar líderes educacionais brasileiros que não via desde a guerra” e retomar suas investigações sobre a nossa educação (Interesse..., 13 jun. 1949). Sua atenção se voltou para o ensino industrial e rural, áreas prioritárias para o Ponto IV, programa de assistência técnica criado pelo governo Truman voltado para a América Latina. A partir da assinatura de acordos bilaterais, o Ponto IV promovia projetos nas áreas de economia, administração, agricultura, transportes, saúde e educação (Damasceno, 2022). Hall se tornaria um grande defensor do programa e do interesse estadunidense em “ajudar” os vizinhos latino-americanos.

Entre 1949 e 1951, Hall foi contratado para atuar no Inep, prestando assessoria técnica ao programa de educação rural coordenado pelo instituto. Entre as atividades desenvolvidas estavam o treinamento de professoras primárias e a produção de uma análise do projeto (Cunha, 2018). A contratação demonstrava o seu reconhecimento como “especialista”, assim como aponta para a construção de vínculos duradouros com o campo educacional brasileiro estabelecidos durante a viagem de 1940 e que perduraram também no contexto de Guerra Fria.

No entanto, sua presença não foi recebida por todos com entusiasmo. Paschoal Lemme, que ajudara Hall em 1940, criticou abertamente a contratação do pesquisador pelo Inep. Após assistir a uma palestra sobre o Ponto IV proferida por Hall no Rio de Janeiro, em 1949, Lemme apontou o “tom de quase arrogância com que se expressava o educador norte-americano, apesar da gentileza no trato pessoal”. Para ele, ao defender as “boas intenções” do governo estadunidense de prestar assistência técnica aos países subdesenvolvidos, Hall estava agindo como “porta-voz do imperialismo americano” (Lemme, 2004, p.25).

As visões de ambos os educadores expressavam o contexto de Guerra Fria, marcado pelas tensões entre anticomunistas e antiamericanos pelo mundo. Segundo Lemme, Hall defendia que, por meio do Ponto IV, os EUA estavam dispostos a “estudar sua assistência técnica e financeira aos governos dos países economicamente subdesenvolvidos, no intuito de cooperar para a elevação do seu padrão de vida” (Hall, 1949, p.48 citado em Lemme, 2004, p.25). Já Lemme enxergava nessa “ajuda” um mecanismo para explorar países mais pobres e mantê-los como “simples produtores de matérias-primas” (p.25).

Percebe-se que, ao longo de sua trajetória, o posicionamento de Hall em defesa da “democracia” vai assumindo novos sentidos. Se, no contexto de guerra, ele criticava o nazismo e a forte centralização do sistema educacional brasileiro, após o conflito, assume uma postura de combate ao comunismo e de defesa do modelo estadunidense. Isso fica evidente no texto “Educación para el desarollo económico”, resultado de uma palestra proferida em Buenos Aires em 1950. Ao tratar do Ponto IV, Hall (1950, p.560) argumentava que “o espírito de ajudar o vizinho menos afortunado é sempre muito forte, particularmente, no povo dos Estados Unidos”. Por isso, eram “infundadas” as acusações de que seu país tivesse interesses imperialistas, reforçando a “boa vontade” de “industriais e do governo” em colaborar com o desenvolvimento de outras nações (p.561). Apesar de se apresentar como um “observador completamente desprovido de caráter oficial”, o que lhe garantiria uma “visão imparcial”, naquele momento sua conexão com os interesses geopolíticos estadunidenses já estava mais do que clara.

Desde 1945, Hall passou a realizar trabalhos de assessoramento para governos e instituições privadas, assumindo papel de consultor em projetos educacionais. Por exemplo, em 1949, foi contratado pelo governo do Irã para auxiliar nas reformas educacionais empreendidas naquele país, chegando a recomendar a adoção do modelo de ensino industrial utilizado nas escolas do Serviço Nacional de Aprendizagem Industrial (Senai) brasileiro. Já em 1955, deixou a Universidade de Columbia para coordenar o treinamento de pessoal da Saudi Arabian Oil Company, permanecendo na Arábia Saudita até os anos 1960.

## Considerações finais

É possível afirmar que, viabilizado pelas redes transnacionais da diplomacia cultural, o reconhecimento de Hall como especialista levou-o a assumir posições importantes, colaborando ou atuando em projetos do governo estadunidense que, por sua vez, convergiam com o de outros países que ele percorreu. No decorrer dessas viagens, definidas (por vezes, de modo não previsto) pelas conjunturas da Segunda Guerra Mundial, Hall atuaria em circuitos transnacionais acadêmicos e também políticos, transitando por espaços geográficos estratégicos para a construção da hegemonia dos EUA.

O caso de Hall ajuda a explicitar a importância que os intercâmbios, oportunizados pela Política da Boa Vizinhança, tiveram na circulação de ideias e de modelos educacionais no continente americano. Esse é um caso que permite observar, conforme vem apontando a historiografia, a complexidade, a multiplicidade de agentes e interesses e as inúmeras contingências que envolvem os processos de circulação de conhecimento entre países.

Os seus estudos sobre a educação latino-americana foram cruciais para sua inserção no campo educacional estadunidense, num momento em que o pan-americanismo era central na política externa dos EUA. Sua entrada nos circuitos da boa vizinhança só foi possível graças ao crescente investimento estatal em políticas de intercâmbio e cooperação que buscavam aproximar estadunidenses e latino-americanos, combatendo a influência do Eixo e promovendo a “amizade” interamericana. Portanto, Hall se beneficiou das possibilidades abertas pela Política da Boa Vizinhança e, ao mesmo tempo, contribuiu diretamente para os interesses que a orientavam.

Suas observações dos imigrantes no Brasil, especialmente dos japoneses, e das medidas do governo Vargas para nacionalizá-los levaram-no a ocupar postos importantes no imediato pós-guerra. Essa foi uma situação particularmente interessante para explicitar como as rotas de circulação são construídas, muitas vezes, por contingências não previstas. Ao mesmo tempo, a trajetória de Hall ajuda a compreender a complexidade e a multiplicidade de interesses e agentes envolvidos na própria construção da diplomacia cultural pública estadunidense, no contexto da Segunda Guerra, quando a América Latina serviu de “laboratório” para a elaboração da sua política externa, e as transformações pelas quais ela passou durante a Guerra Fria, assumindo novos contornos e interesses que também impactaram nos objetos de estudo e na atuação do pesquisador.

## Data Availability

Não estão em repositório.

## NOTAS

1 Nascido em Kewanee, Illinois, Robert King Hall era descendente de irlandeses presbiterianos que emigraram para os EUA no final do século XVII. Estudou na Deerfield-Shields High School e graduou-se com honras em física e matemática pela Lake Forest University, em 1934. Mestre em física pela Universidade de Harvard (1935) e em educação pela Universidade de Chicago (1936). Doutor em educação comparada pela Universidade de Michigan (1941). Entre 1936 e 1941, foi professor da Cranbrook School, uma escola privada para rapazes em Michigan. Com a entrada dos EUA na guerra, alistou-se na Marinha e frequentou a Naval School of Military Government and Administration at Columbia University e, em seguida, tornou-se assistant academic director of the Naval School of Military Government at Princeton University. Entre 1945 e 1946, atuou em missão no Japão, a serviço do governo estadunidense. Em 1947, passou a integrar o corpo docente do Teachers College da Universidade de Columbia. Como consultor técnico, colaborou com o Instituto Nacional de Estudos Pedagógicos entre 1949 e 1951. Em 1949, prestou assessoria ao governo do Irã. E, em 1955, passou a atuar no treinamento de funcionários da Arabian American Oil Company. Retornou aos EUA nos anos 1960 e continuou trabalhando como técnico para empresas e instituições, a exemplo do College of Petroleum & Minerals da Arábia Saudita.

2 No início do século XX, especialmente no pós-Primeira Guerra Mundial (1914-1917), diversas instituições filantrópicas, como as fundações Carnegie e Rockefeller, investiram no patrocínio de intercâmbios científicos e culturais como forma de promover as relações internacionais. Acreditava-se ser possível manter a paz entre as nações por meio do entendimento mútuo. Ver, entre outros: Batista (2024); Batista, Ferreira (2021); Palmer (2015).

3 Esta e as demais citações em língua inglesa têm tradução livre.
